# Revealing the Potential of Star Anise Essential Oil: Comparative Analysis and Optimization of Innovative Extraction Methods for Enhanced Yield, Aroma Characteristics, Chemical Composition, and Biological Activities

**DOI:** 10.1002/fsn3.4508

**Published:** 2024-10-18

**Authors:** Guangjie Zhang, Zheng Ma, Yan Piao, Ning Li, Canfeng Bian, Chenxi Ren, Mingyuan Gao, Weiming Yue, Tianzhu Guan

**Affiliations:** ^1^ School of Biology and Food Engineering Anyang Institute of Technology Anyang China; ^2^ Department of Thoracic Surgery Qilu Hospital of Shandong University Jinan China; ^3^ Department of Intensive Care Unit Jilin Heart Hospital Changchun China; ^4^ School of Food Science and Engineering Yangzhou University Yangzhou China

**Keywords:** aroma properties, biological activity, essential oil extraction, process optimization, star anise

## Abstract

Star anise has been used for a long time in improving human health and curing diseases, owing to its unlimited components with complex chemical structures and a wide range of bioactivities. This study is aimed to investigate the influence of extraction methods (steam distillation, ethanol Soxhlet extraction, supercritical carbon dioxide extraction, subcritical n‐butane extraction) on the yield, aroma properties, chemical composition, and bioactivity of star anise essential oils. Electronic nose detection revealed the essential oils from subcritical extraction exhibited the most intense aroma, while the essential oils from ethanol Soxhlet extraction had a more complex aroma profile. Fourier‐transform infrared analysis showed the presence of benzene rings, carbonyl groups, C=C, and aromatic ether bonds in the essential oils extracted through different methods. The major components were heterocyclic olefins, heterocyclic oxygenates, and aromatic oxygenates, as well as certain amounts of flavonoids and polyphenols. Correlation analysis revealed the relative contents of volatile *trans*‐anethole, wormwood, d‐limonene, cineole, and *trans*‐α‐citronelene were strongly associated with the antibacterial activity of the essential oils. Similarly, the contents of volatile components (d‐limonene, cineole) and non‐volatile components (total flavonoids and total polyphenols) were strongly correlated with the DPPH scavenging activity of the essential oils. These results confirm the effectiveness of the ethanol Soxhlet extraction method in retaining the bioactivity of the essential oils. Finally, with a Box–Behnken central composite design of response surface and single‐factor experiments, the optimal extraction conditions for the ethanol Soxhlet method were determined: ultrasonic frequency of 80 kHz, crushing particle size of 60 meshes, liquid–material ratio of 8:1 (mL:g), and ultrasonic time of 35 min. Under these conditions, the essential oil yield was 25.51% ± 0.21%. Overall, these findings highlight the significance of extraction methods in obtaining high‐quality star anise essential oils with desirable aroma properties and potent bioactivities.

## Graphical abstract

This work aimed to investigate the influence of different extraction methods (steam distillation, ethanol Soxhlet extraction, supercritical carbon dioxide extraction, subcritical n‐butane extraction) on the yield, aroma properties, chemical composition, and bioactivity of star anise essential oils. FT‐IR, electronic nose, and correlation analysis had been proved to be extremely insightful for the strongly correlated with the DPPH scavenging activity of the essential oils under the optimal extraction conditions.

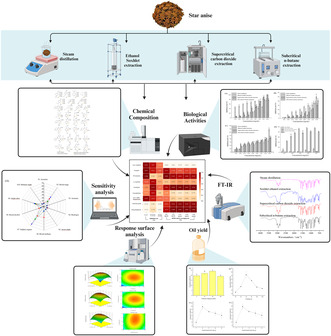

## 1 Introduction

Medicinal and edible plants have been applied for centuries as remedies for human diseases and offer an outstanding source of bioactive constituents in traditional Chinese medicine (Guan et al. 2022; Sun‐Waterhouse et al. 2024). The dried fruits of *Illicium verum* Hook. F., commonly known as Chinese star anise, refer to the dried fruits of a woody species that are extensively utilized and endemic to Southeast China and North Vietnam (Guan et al. 2021). From recent phytochemical and pharmacological studies, the raw material star anise has a volatile content of 2.5%–3.5% in fresh fruits and 8%–9% in dried materials with potential biological activities (Loi and Thu 1970). The main component in star anise essential oils is *trans*‐anethole, a paramethoxyphenyl propene that accounts for 85%–90% by weight. The rest components include terpene, pinene, β‐phellandrene, α‐diterpene, limonene, estragole, safrol, and terpineol (Chen et al. 2023). Especially, Chinese star anise serves as a valuable industrial source of shikimic acid, a key component used to produce therapeutic agents that can efficiently mitigate the severity of the influenza virus (Avula et al. 2009). Consequently, there is a growing interest in utilizing star‐anise‐derived bioactive compounds as functional ingredients in various applications, such as foods, bakery products, beverages, and cosmetics (Ren et al. 2024). These natural ingredients possess potential health benefits, including anti‐carcinogenic, anti‐hypertensive, antimutagenic, antimicrobial, and antioxidant activities.

The composition, content, quality, and bioactivity of star anise essential oils vary depending on the solvents and extraction techniques employed (Li et al. 2025; Plati and Paraskevopoulou 2022). Consequently, efficient systems are needed to evaluate medicinal plants as antimicrobial agents based on evidence‐based activity results (Arcot et al. 2024). The various commonly‐used extraction techniques include steam distillation, Soxhlet ethanol extraction, supercritical carbon dioxide extraction, subcritical n‐butane extraction, and their combinations. However, these traditional techniques often require long extraction times, posing a risk of thermal degradation for many phytoconstituents (Kumar et al. 2023). Furthermore, these solvent‐based extraction methods rely on the proper choice of solvents and the use of heat or agitation to improve solubility and mass transfer. Whether these extraction methods effectively extract the bioactive components from star anise and other medicinal plants remains uncertain, given the diversity of plant species and their constituents. In particular, the efficiency of these methods in extracting antioxidants from medicinal plants is not well‐known (Guan et al. 2021). Therefore, further research is needed to optimize the extraction techniques and evaluate their effectiveness in capturing the full‐spectrum of bioactive compounds in star anise and other herbal ingredients.

As a part of our ongoing research, we hypothesize that different solvents may influence the content and quality of phenols in star anise, thereby affecting its bioactivity potential (Viacava, Cenci, and Ansorena 2022). Despite ample literature on the chemical, antibacterial, and insecticidal properties of star anise, there is no information regarding the antioxidant properties of its volatile oils and acetone extracts (Liu, Tao, and Huang 2021). Herein, we comprehensively studied the chemical compositions and yields of star anise essential oils obtained through different extraction methods and systematically compared the chemical properties and yield of the extracts. Sensory evaluation methods were employed to compare the aroma properties of the star anise essential oils extracted using different methods and to compare the properties of different oils (El‐Shamy and Farag 2022). Furthermore, the bioactivities of star anise essential oils were investigated, and the correlation between the main component contents and the bioactivity of the oils extracted by different methods was analyzed. Lastly, our research was aimed at optimizing the star anise essential oil extraction techniques through single‐factor experiments and response surface design.

Overall, our research was aimed at providing comprehensive insights into the extraction methods and properties of star anise extracts, especially the antioxidant, antimicrobial, and sensory properties. This study will help better understand the potential applications of star anise in various fields, including food science and medicinal research.

## 2 Materials and Methods

### 2.1 Materials

Chinese yam was purchased from a local supermarket in Anyang. *Rhizopus stolonifera*, *Escherichia coli*, and *Saccharomyces cerevisiae* were provided by the Anyang Institute of Technology. 1,1‐Diphenyl‐2‐trinitrophenylhydrazine (DPPH, 97%, 1898‐66‐4), tryptic soy broth (TSB, T770421), and sabouraud dextrose broth (SDB, 771981) were purchased from TCI (Shanghai) Chemical Industry Development Co., Ltd. All other reagents were of analytical grade.

### 2.2 Sensory and Yield Evaluation of Star Anise Essential Oil in Diverse Methods

For extraction of essential oils, star anise was crushed and screened through a 60‐mesh sieve after being oven‐dried overnight at 40°C. The steam distillation, ethanol Soxhlet extraction, supercritical carbon dioxide extraction, and sub‐critical n‐butane extraction methods were further analyzed using sensory evaluation and extraction rate. The yield (*Y*, %) of star anise essential oils was calculated as follows:

(1) $$Y=\frac{m_{1}}{m_{0}}\times 100\%$$

where *m*_1_ is the quantity of star anise essential oils obtained after drying or rotary evaporation (g), and *m*_0_ is the quantity of raw star anise (g).

### 2.3 Aroma Property Evaluation of Star Anise Essential Oils

The aroma properties of star anise essential oils extracted by different methods were determined using a PEN3 electronic nose (Schwerin, Germany) with 10 gas sensors and by calculating the resistance ratio of volatile gas to the blank (*G*/*G*0). Briefly, 200 μL of essential oils extracted from each method were placed in a 20 mL vial and stabilized at room temperature for 30 min. The parameters for the PEN3 were set as follows: detection time of 1 min, gas flow rate of 400 mL/min, waiting time of 5 s, and washing time of 90 s. All samples were analyzed in triplicate and compared in terms of differences via principal component analysis (PCA).

### 2.4 Comparison of Star Anise Essential Oils

The components of star anise essential oils were produced as previously described (Li et al. 2024). Briefly, 5 mL of a star anise essential oil sample was dissolved in anhydrous ethanol. After ultrasonication and filtering, the compositions were detected via gas chromatography—mass spectrometry (GC–MS) with a J&W122‐5532 quartz capillary column (30 m × 250 μm × 0.25 μm). The MS conditions were as follows: an ion source with electron impact and at 230°C; an MS quadrupole temperature of 150°C; a mass scan range of 30–500 amu.

### 2.5 Fourier‐Transform Infrared (FT‐IR) Spectroscopy

FT‐IR spectra of the star anise essential oils were measured according to a previous work (Gao et al. 2022) with minor modifications. Briefly, dried KBr (100 mg) was ground and compressed into tablets. Then a star anise essential oil sample extracted with a certain method was added onto the KBr tablets and analyzed using a Bruker Tensor II FT‐IR spectrometer (Ettlingen, Germany). The spectral data were collected under identical conditions from 4000 to 500 cm^−1^ at room temperature.

### 2.6 Analysis of Total Flavonoid and Total Polyphenols Contents

#### 2.6.1 Total Flavonoid Content

The 0.196 mg/mL rutin dissolved in 60% alcohol was chosen as the standard solution. Then the standard solution (0, 1, 2, 3, 4, and 5 mL) was mixed with 1 mL of 10% sodium nitrite, and after standing for 6 min, was added with 1 mL of 10% aluminum nitrate and 10 mL of 4% NaOH. Next, the absorbance at 415 nm was determined spectrophotometrically after 15 min of standing. A linear regression equation between absorbance and the standard solution concentration was established to determine the total flavonoid content in star anise essential oil samples.

#### 2.6.2 Total Phenolic Content

Gallic acid (51.5 mg, ≥ 99%) was accurately weighed and dissolved in 50 mL of 60% ethanol to obtain a 1.0197 mg/mL standard solution. Then 0.2, 0.5, 1.0, 1.5, or 2 mL of the standard solution was mixed with 5 mL of 10% Folin phenol as the reagent and 4 mL of 7.5% Na_2_CO_3_. After incubation for 60 min at room temperature, the absorbance at 765 nm was measured. Based on the standard curve of total phenolic concentration, 1 mL of star anise essential oils was diluted with 60% ethanol and repeated to determine the total phenolic content.

### 2.7 Comparison of Bioactivities of Star Anise Essential Oils

#### 2.7.1 Comparison of Antibacterial Activity

To prepare the culture medium, certain amounts of tryptic soy agar (TSA), TSB, sabouraud dextrose agar (SDA), and SDB were weighed and mixed with water according to the instructions. The mixture was sterilized at 121°C/20 min and allowed to cool under aseptic conditions. TSA and SDA were poured into plates and left to solidify at 45°C. *R. stolonifera*, *S. cerevisiae*, or *E. coli* was transferred to the inclined plane of a test tube and activated for 24 h. Two to three rings of each culture were then inoculated into the liquid culture medium and shaken at 150 rpm/min for 12 h. With a sterile pipette, 1 mL of the resulting bacterial suspension was mixed with 9 mL of sterile water. This mixture was then repeated, and 1 mL of the diluted bacterial suspension was taken and mixed with 9 mL of sterile water. Double dilution was continued through analogy. The reference was consulted for further guidance. The bacterial suspension with a bacterial count of about 105 cfu/mL was collected using a blood cell counting plate method (*R. stolonifera* and *S. cerevisiae*) or a plate counting method (*E. coli*). For *R. stolonifer* and *S. cerevisiae*, bacterial suspensions were prepared and antibacterial performances were detected at 28°C, and the media were SDA and SDB. However, for all cultures involving *E. coli*, the temperature was 37°C, and the media were TSA and TSB.

Bacteriostatic performances were determined using the inhibition rate method on a microtiter plate (Wang et al. 2011) with some slight modifications. To compare and analyze the bacteriostatic performances of star anise essential oils before and after inclusion, 1 mL of a bacterial suspension was inoculated into 150 mL of a liquid medium (SDB for *R. stolonifer* and *S. cerevisiae*, TSB for *E. coli*). Under aseptic conditions, 300 μL of an inoculation solution containing essential oils extracted with a certain method was injected into each well, and the concentrations of essential oils were controlled at 0, 0.01, 0.05, 0.10, 0.20, and 0.40 mg/mL. The wells were then covered and placed in an automatic analyzer, and microbial growth curves were observed after 24 h. Next, the microbial inhibition rate (*I*, %) was calculated as follows:

(2) $$I=\frac{\Delta {\mathrm{OD}}_{C}-\Delta {\mathrm{OD}}_{S}}{\Delta {\mathrm{OD}}_{C}}\times 100\%$$

where ΔOD_C_ and ΔOD_S_ are the changes in the optical density (OD) at 600 nm of the positive control culture medium and the sample culture medium respectively added with essential oils or inclusion compounds after a certain period of culture.

The minimum inhibitory concentration (MIC) of the star anise essential oils extracted with each method was determined using the microdilution method (Rakmai et al. 2017). The MIC was recorded as the concentration of essential oils that did not result in a significant increase in OD (ΔOD_600_ ≤ 0.05) in the culture solution within the well after 24 h, as per the previously described antibacterial performance test method.

#### 2.7.2 Comparison of DPPH Radical Scavenging Activity

The DPPH free radical scavenging activity was assessed in accordance with the manufacturer's instructions (Guan et al. 2024). A certain amount of DPPH was weighed accurately, dissolved in anhydrous ethanol, and transferred to a 100 mL volumetric flask. The volume was diluted to 100 mL to form a 0.1 mmol/L DPPH solution. Star anise essential oils, extracted by each of the four methods, were diluted with anhydrous ethanol to different concentrations (0.01, 0.05, 0.1, 0.2, 0.4 mg/mL) to obtain the test solutions.

For the DPPH radical scavenging assay, 2 mL of anhydrous ethanol and 2 mL of the DPPH solution were mixed in a colorimetric tube and shaken to record the initial absorbance (*A*_0_). Then 2 mL of the test solution and 2 mL of the DPPH solution were mixed in another colorimetric tube and shaken to record the absorbance (*A*_*i*_). Finally, 2 mL of the test solution and 2 mL of anhydrous ethanol were mixed in a new colorimetric tube and shaken to record the absorbance (*A*_*j*_). The three tubes were kept in the dark for 30 min, and their absorbance values were measured at 517 nm. The DPPH clearance rate (CR, %) was calculated using Equation (3):

(3) $$\mathrm{CR}=\left(1-\frac{A_{i}-A_{j}}{A_{0}}\right)\times 100\%$$

#### 2.7.3 Correlation Analysis Between Main Component Contents and Bioactivity of Star Anise Essential Oils Extracted With Different Methods

The differences in the contents of volatile components, total flavonoids, and total polyphenols in star anise essential oils extracted from different methods result in variations in bioactivities. Several scholars have studied the components responsible for the differences in the bioactivities of star anise essential oils and mostly suggest the association with the volatile components. To further explore the reasons for the different bioactivities of star anise essential oils extracted from different methods, the relative contents of common volatile components, total flavonoids, and total phenolic acid in star anise essential oils, and their correlation with antibacterial activity and DPPH‐free radical scavenging activity were analyzed. The inhibitory rate (%) of each essential oil extract on three tested microorganisms at the concentration of 6.4 mg/mL was selected as the reference index.

### 2.8 Optimization of Extraction Technology of Star Anise Essential Oil

The bioactivity of star anise essential oils obtained from ethanol Soxhlet extraction was the highest among the extraction methods. Ultrasonic treatment was conducted before extraction to improve production. Additionally, the extraction technology was optimized using single‐factor experimentation and response surface design.

#### 2.8.1 Single‐Factor Experimentation

The ultrasound frequency, crushing size, liquid–material ratio, and ultrasonic time were optimized on the basis of the yield of star anise essential oils to improve production.

- Ultrasound frequency (kHz): The crushing size was set at 60 meshes, and the liquid–material ratio was 1:15. The yield of star anise essential oils was tested under ultrasound frequencies of 0, 45, 80, and 100 kHz and ultrasonic time of 25 min.
- Particle size (mesh): Under the optimal ultrasonic frequency, the liquid–material ratio was 1:15 and the ultrasonic time was 25 min. The yield of star anise essential oils was tested at the crushing sizes of 20, 40, 60, 80, and 100 meshes.
- Material–liquid ratio (g:mL): Under the optimal ultrasonic frequency, the crushing size was set at 60 meshes and the ultrasonic time was 25 min. The yield of star anise essential oils was tested at a liquid–material ratio of 1:5, 1:10, 1:15, 1:20, and 1:25.
- Ultrasonic time (min): Under the optimal ultrasonic frequency, the crushing size was set at 60 meshes and the liquid–material ratio was 1:15. The yield of star anise essential oils was tested at ultrasonic times of 5, 15, 25, 35, and 45 min.

#### 2.8.2 Response Surface Design

According to the single‐factor experiments, crushing size, material–liquid ratio, and ultrasonic time were optimized using three‐factor, three‐level Box–Behnken design and response surface design. The factor coding and levels were shown in Table S1. Results were processed and analyzed using Design‐Expert 8.0.5.

## 3 Results and Discussion

### 3.1 Sensory and Yield Evaluation

Due to the differences in solvents, temperature, and pressure, the compositions of star anise essential oils were diverse, resulting in various sensory characteristics. The specific technological parameters of each extraction method are presented in Table 1. The star anise essential oils extracted from steam distillation are lighter in color compared with other methods, while star anise essential oils obtained through supercritical carbon dioxide extraction have similar color to those from sub‐critical n‐butane extraction (Table 1). In terms of odor, a strong anethole was found in all the extracted samples. Also, anethole had significantly affected the perception of other volatile constituents, thus, the subsequent studies, such as correlation analysis, are highly necessary. From the perspective of yield, the yield from ethanol Soxhlet extraction is the highest (10.86 ± 0.83, *p* < 0.05). The yields of steam distillation, supercritical carbon dioxide extraction, and sub‐critical n‐butane extraction methods are all higher than in previous studies, which may be due to the raw material processing, extraction process, solvent recovery, and treatment methods.

**TABLE 1:** The sensory analysis of star anise essential oil extracted by different methods.

Methods	Steam distillation	Ethanol soxhlet extraction	Supercritical carbon dioxide extraction	Subcritical n‐butane extraction
Appearance	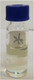	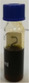	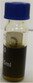	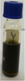
Color	Colorless to pale yellow	Dark brown with a yellow‐greenish hue	Deep brown with a greenish undertone	Deep brown with a greenish undertone
Smell	Strong anethole aroma	Intense star anise aroma with a prominent ethanol note	Strong anethole aroma	Strong anethole aroma
Yield *Y* (%)	10.86 ± 0.83^b^	21.84 ± 1.44^a^	7.88 ± 0.17^c^	7.77 ± 0.31^c^

*Note:* Different letters in the yield indicate significant differences. (*p* < 0.05).

### 3.2 Aroma Properties of Star Anise Essential Oils

**FIGURE 1:**
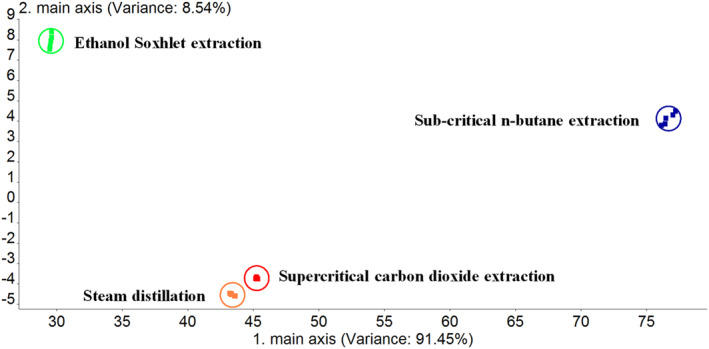
The principal component analysis of aroma components of star anise extracts extracted by different methods.

**FIGURE 2:**
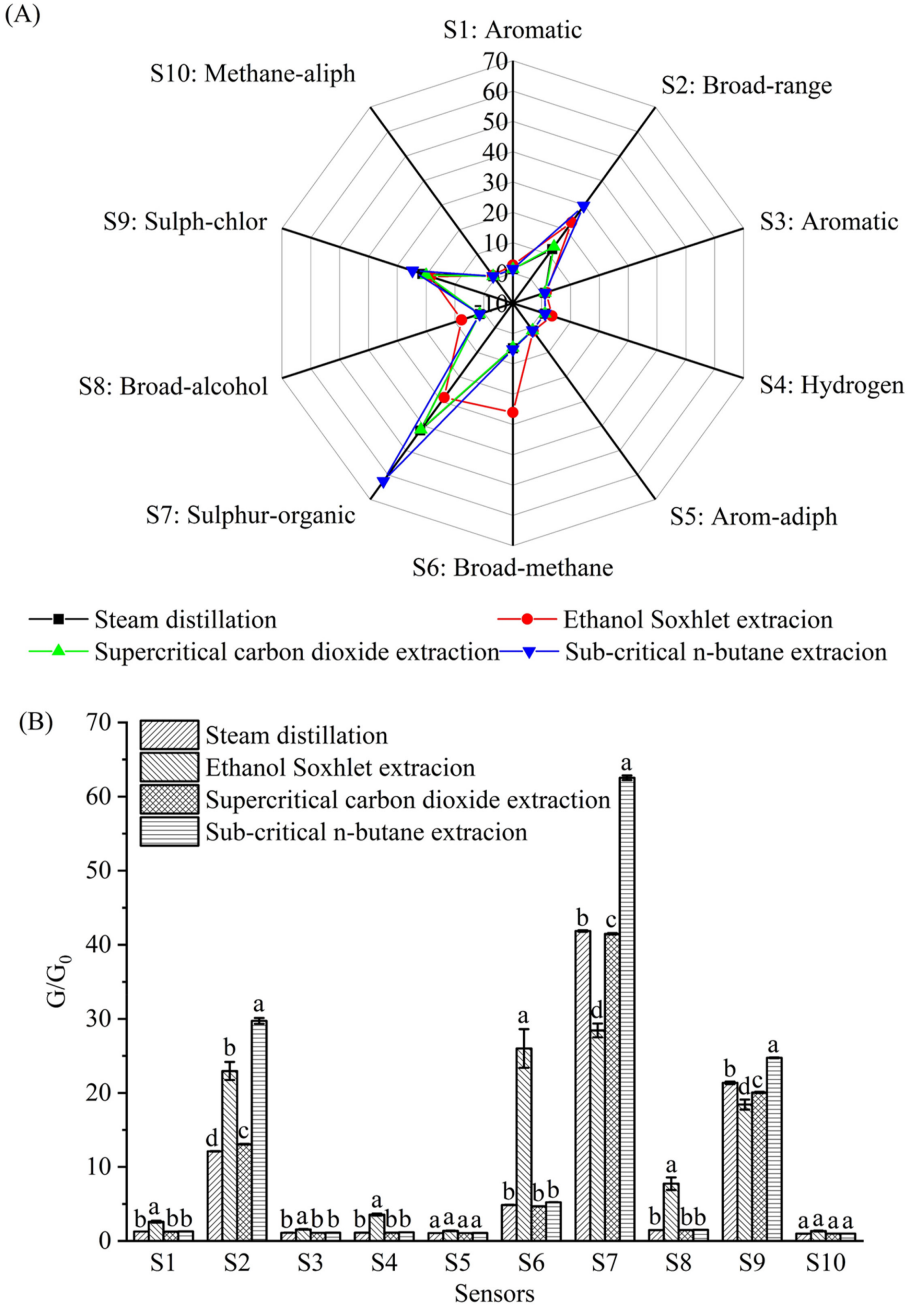
Radar chart (A) and bar chart (B) for sensitivity analysis of star anise extracts extracted by different methods to different sensors of the electronic nose.

An electronic nose was adopted to identify the differences in the star anise essential oils obtained from various extraction methods. The contribution rates of the first and second principal component variances are 91.45% and 8.54%, respectively (Figure 1). The cumulative contribution rate reaches 99.95% (> 85%), which may represent the overall information of the four samples. Except steam distillation and supercritical carbon dioxide extraction, the aroma properties between the samples from the other methods significantly differ, indicating there are a few differences among various extraction methods. However, the difference in aroma properties of the samples extracted via steam distillation and supercritical carbon dioxide is relatively slight. Further observation shows from Figure 2 that the star anise essential oils obtained from all four methods had higher sensitivity to sensors S2, S7, and S9, especially for the extract from sub‐critical n‐butane. The odor properties corresponding to these sensors were “wide‐ranging”, “organic sulfur”, and “sulfur‐chlorine”, which may be related to the strong irritant volatile components of star anise essential oils. Additionally, the star anise essential oils obtained from ethanol Soxhlet extraction showed more abundant aroma properties, and were more sensitive to sensors S1, S4, S6, and S8 than other methods. This result indicates the essential oils obtained from this method also have “aromatic”, “hydrogen”, and “obvious methane and ethanol odor” in addition to the odor properties of “wide‐ranging”, “organic sulfur”, and “sulfur‐chlorine”. The differences may be ascribed to the solvent residues in essential oils and distinctions of components.

### 3.3 GC–MS Analysis of Star Anise Essential Oils

**FIGURE 3:**
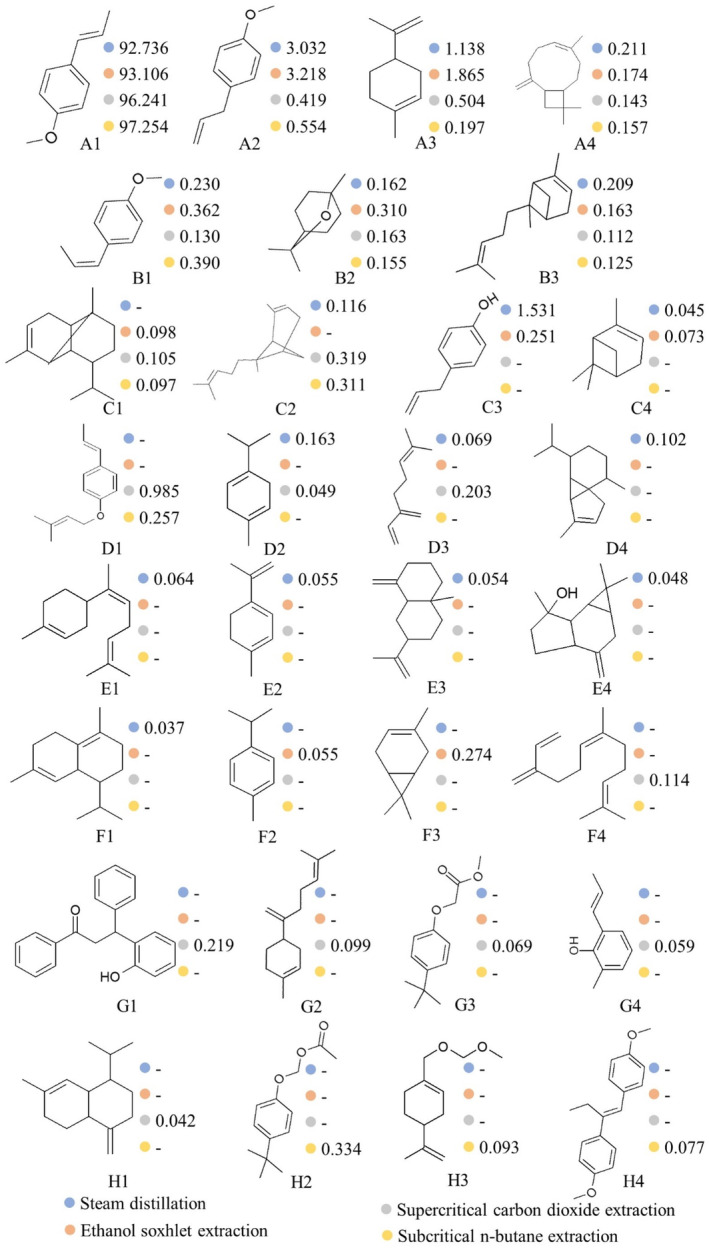
The GC–MS analysis of the star anise extracts extracted by four different methods, including steam distillation (blue), ethanol Soxhlet extraction (orange), supercritical carbon dioxide extraction (gray) and subcritical n‐butane extraction (yellow). A1, *trans*‐Anethole; A2, Estragole; A3, d‐Limonene; A4, Caryophyllene; B1, *cis*‐Anethole; B2, Eucalyptol; B3, *trans*‐.alpha.‐Bergamotene; C1, Copaene; C2, *cis*‐.alpha.‐Bergamotene; C3, 4‐(2‐Propenyl)‐phenol; C4, .alpha.‐Pinene; D1, 1‐(3‐Methyl‐2‐butenoxy)‐4‐(1‐propenyl)benzene; D2, .gamma.‐Terpinene; D3, beta.‐ Myrcene; D4, alpha.‐Cubebene; E1, *cis*‐.alpha.‐Bisabolene; E2, 1,3,8‐p‐Menthatriene; E3, beta.‐Selinene; E4, (−)‐Spathulenol; F1, delta.‐Amorphene; F2, *p*‐Cymene; F3, (+)‐3‐Carene; F4, *cis*‐.beta.‐Farnesene; G1,3‐(2‐Hydroxyphenyl)‐1,3‐diphenyl‐1‐propanone; G2, beta.‐Bisabolene; G3, Acetic acid, [4‐(1,1‐dimethyl‐lethyl)phenoxy]‐,methyl ester; G4, 2‐(1‐Propenyl)‐6‐methylphenol; H1, .gamma.‐Muurolene; H2, [4‐(1,1‐dimethyl‐ethyl)phenoxy]‐methyl acetate; H3, 4‐Isopropenyl‐1‐methoxymethoxymethyl‐cyclohexene; H4, 1,1′‐(1‐Ethyl‐1,2‐ethenediyl) bis(4‐methoxybenzene). The bold number after the legend indicates that the component was detected in all extracts obtained by the four extraction methods, while the dashed line indicates that the component was not detected in the extract obtained by that extraction method.

All chemical components identified by GC–MS are summarized in Figure 3. There are 18, 12, 18, and 13 independent components corresponding to steam distillation, ethanol Soxhlet extraction, supercritical carbon dioxide extraction, and sub‐critical n‐butane extraction, respectively. These results differ from a previous study (Yan, Xiao, and Huang 2002), which may be attributed to differences in raw materials and GC–MS conditions. Seven common components were found in all four types of essential oils, including d‐limonene, eucalyptol, camphor, cis‐anethole, *trans*‐anethole, caryophyllene, and *trans*‐α‐citronelene. However, their relative contents vary. *Trans*‐anethole accounting for more than 90% is the main component. The essential oils from subcritical extraction have the highest relative content of *trans*‐anethole (97.254 ± 0.035%), which may be the main reason why all essential oil samples exhibit a strong anise aroma and slightly differ in the electronic nose signals.

### 3.4 FT‐IR Analysis

**FIGURE 4:**
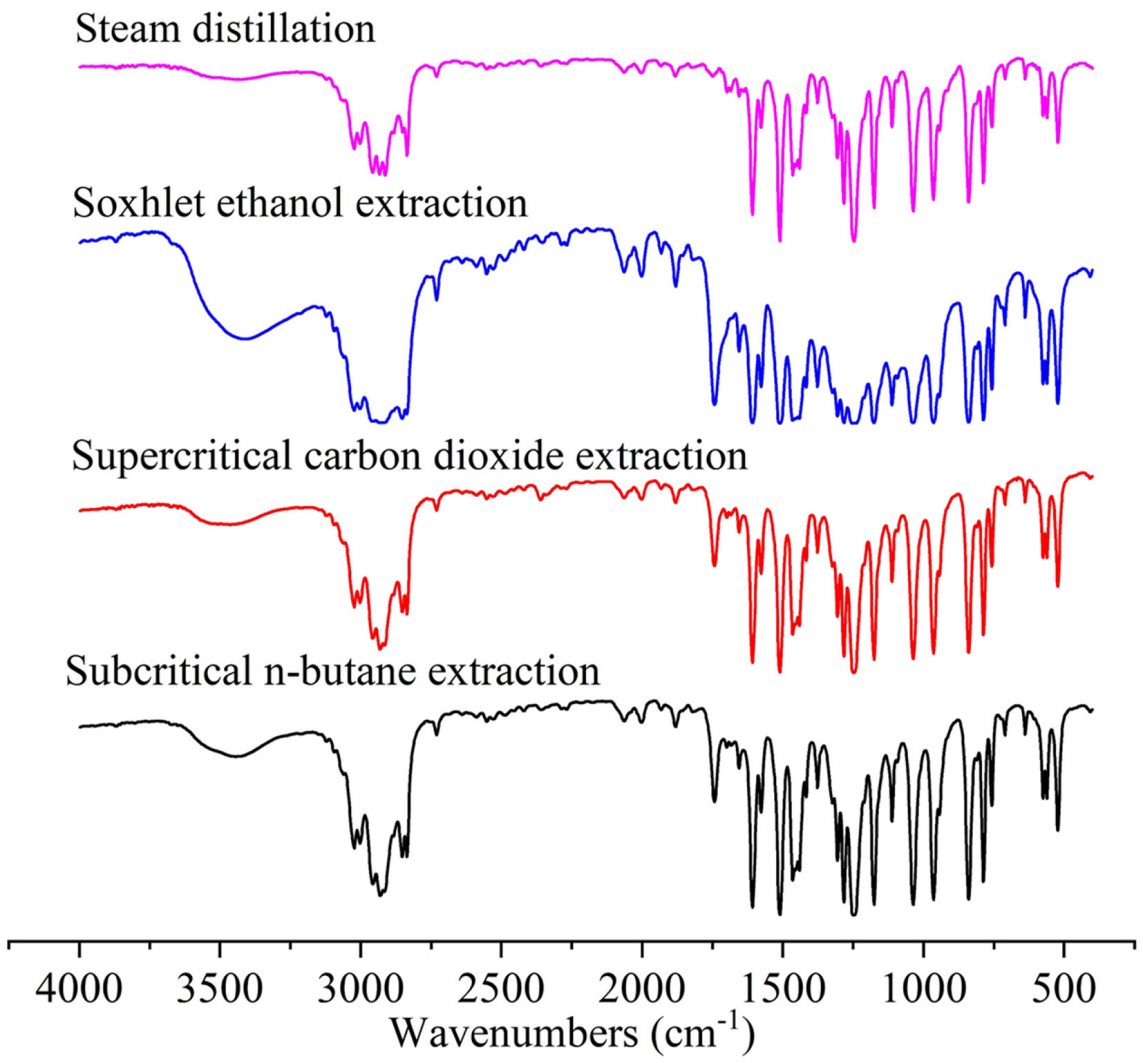
FT‐IR spectra of star anise extracts extracted by different methods.

The FT‐IR spectra of the four types of essential oils are broadly similar (Figure 4), which may be due to the common functional groups of similar compositions. In detail, the ethanol Soxhlet extracted oils mainly differ from other samples in the absorption peak near 3400 cm^−1^ (–OH stretching vibration), which may be ascribed to the presence of phenolic substances or residual ethanol. In addition, molecular association causes a slight redshift and enhances the spectral width and strength. The peaks near 3020 and 3000 cm^−1^ reflect the C–H stretching vibration of the benzene ring and C=C. The peaks near 2930 and 2840 cm^−1^ stand for the C–H stretching vibration of methyl and methylene.

The essential oils from steam distillation had a quite weak absorption peak near 1747 cm^−1^, where representing the C=O stretching vibration of carbonyl group. However, different from essential oils from steam distillation, the other three types of essential oils with representative peaks at 1747 cm^−1^ correspond to the stretching vibration of carbonyl group. It can be speculated that the oils from steam distillation are clear and transparent in appearance, but the colors of the other three types of essential oils are dominated by colored ketones and esters (e.g., flavonoids and anthraquinones). The four sharp peaks at 1607, 1510, 1464, and 1442 cm^−1^ correspond to the stretching vibration of the benzene ring. The four peaks near 1285, 1246, 1176, and 1033 cm^−1^ imply the C–O stretching vibration of the aromatic ether bond. The peak near 963 cm^−1^ reflects the in‐plane bending vibration of C–H on the *trans*‐C=C, and the C–H bending vibration peaks of substituted aromatic compounds appear at 839 and 784 cm^−1^. Since the relative contents of *trans*‐anethole in star anise essential oils extracted from different methods are all above 90%, the spectra of the essential oils in Figure 4 are extremely alike to some reported spectra (Geraldes et al. 1995). The spectra indicate that star anise essential oils contain groups such as benzene ring, carbonyl, C=C, and aromatic ether bond, which is consistent with the GC–MS results in Section 3.3.

### 3.5 Total Flavonoid and Total Polyphenol Contents

**FIGURE 5:**
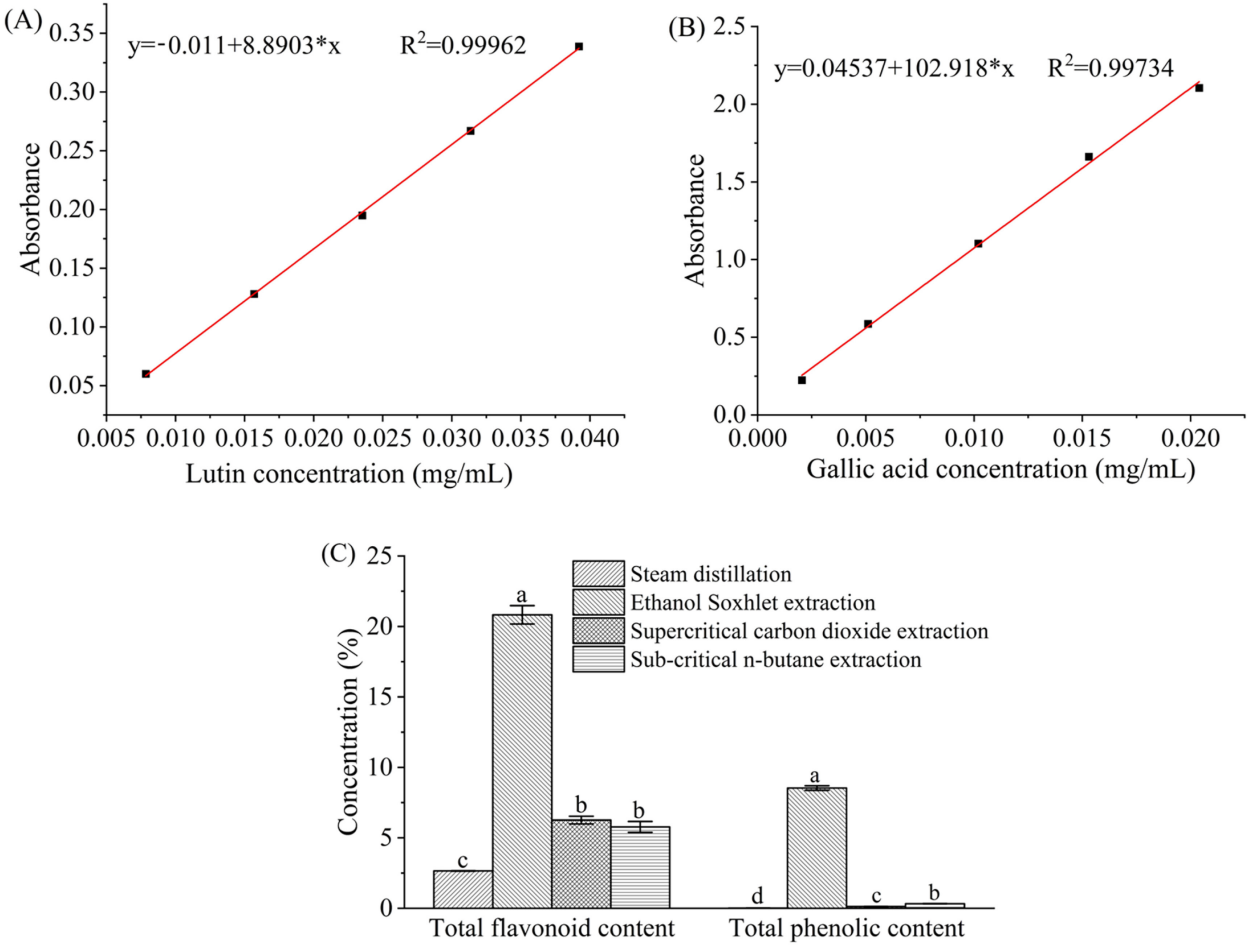
The standard curves of rutin (A) and gallic acid (B), as well as the comparison of total flavonoids and total polyphenols in the star anise extracts extracted by different methods (C).

From a health‐benefit perspective, there is significant scientific interest in plant‐derived total flavonoids and polyphenols, which are produced as antioxidant and protective compounds to exert benefit effects on human health owing to their activities against environmental stresses. Rutin and gallic acid were adopted as reference materials to measure the total flavonoid and polyphenol contents in the star anise essential oils extracted from various methods. The standard curves for rutin and gallic acid are illustrated in Figure 5A,B. Owing to the strong linear relationship, the regression equations for the standard curves of rutin and gallic acid, *Y* = 8.8903*X*−0.0113 (*R* ^2^ = 0.9996) and *Y* = 102.9181*X* + 0.0454 (*R* ^2^ = 0.9973), can be utilized to accurately predict the total flavonoid and polyphenol contents in the essential oils. The total flavonoid and total polyphenol contents in the essential oils from ethanol Soxhlet extraction are significantly higher compared with other methods (*p* < 0.05, Figure 5C). The contents of total flavonoids and total polyphenols are 20.83 ± 0.65% and 8.53 ± 0.16%, respectively, which is consistent with two previous studies (Aly et al. 2016; Padmashree et al. 2007). Also, the total flavonoid and total polyphenol contents in the ethanol Soxhlet extracted essential oils are higher than the results from Soher E. Aly (65.9 ± 0.9 and 112.4 ± 0.95 mg/g, respectively) and from A. Padmashree et al. (6823 ± 18 and 2950 ± 21 ppm, respectively) (Padmashree et al. 2007).

### 3.6 Bioactivities of Star Anise Essential Oils

#### 3.6.1 Antibacterial Activity Investigations

**FIGURE 6:**
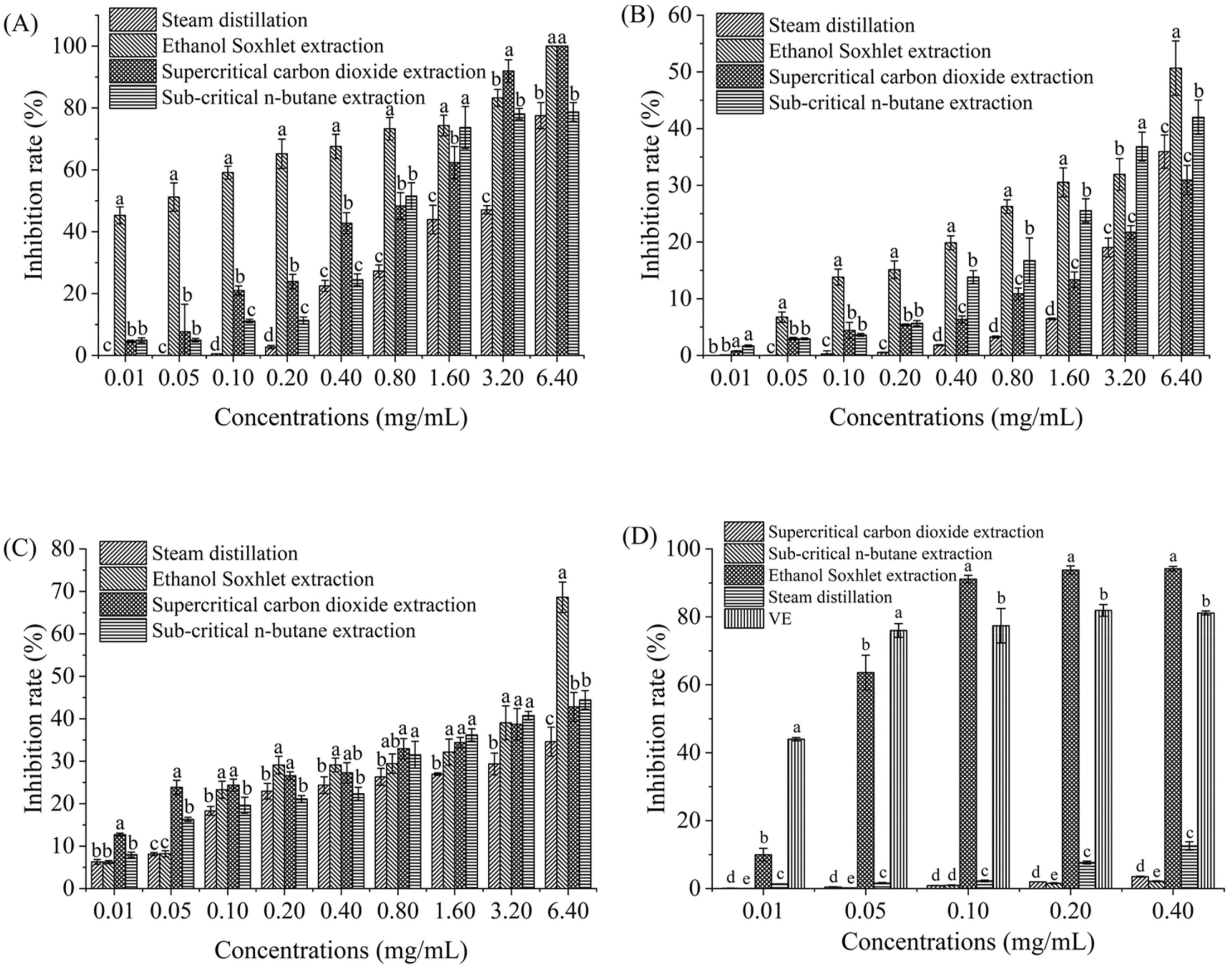
The inhibition effect of star anise extracts on *Rhizopus stolonoifer* (A), *Saccharomyces cerevisiae* (B), and *E. coli*. (C), as well as the DPPH radical scavenging effect of the star anise essential oil (D). Different letters indicate significant differences in the data. (*p* < 0.05).

The four types of star anise essential oils all have inhibitory effects on the three tested microorganisms to varying degrees (Figure 6). The ethanol Soxhlet extracted essential oils have a significantly inhibitory effect on *R. stolonifera* and *S. cerevisiae* compared with other essential oils (*p* < 0.05, Figure 6A,B). The inhibitory effects of the four types of essential oils on *E. coli* are not significantly different (0.01–3.20 mg/mL), but the inhibitory effect of the star anise essential oils extracted by ethanol Soxhlet at 6.40 mg/mL is better. Currently, methanol, ethanol, and acetone are widely utilized as polar solvents to extract flavonoids and polyphenols. Compared with other solvents, ethanol is a more satisfying choice and is safer for human consumption. Furthermore, the results of MIC on antimicrobial activities underlie the current research on cyclodextrin inclusion complex investigations (Figure 6C). MIC was undetectable within the tested concentration range under most conditions (Table S2). This test was aimed at selecting the essential oils with broad‐spectrum bacteriostatic activity and high comprehensive activity as the active ingredient for the subsequent cyclodextrin inclusion experiment and composite membrane preparation. Therefore, despite the small values of essential oils in the subsequent cyclodextrin inclusion complex, we did not further expand the concentration range to test MIC. In all, the ethanol‐Soxhlet‐extracted essential oils have broad‐spectrum antibacterial activity.

#### 3.6.2 DPPH Radical Scavenging Activity

**FIGURE 7:**
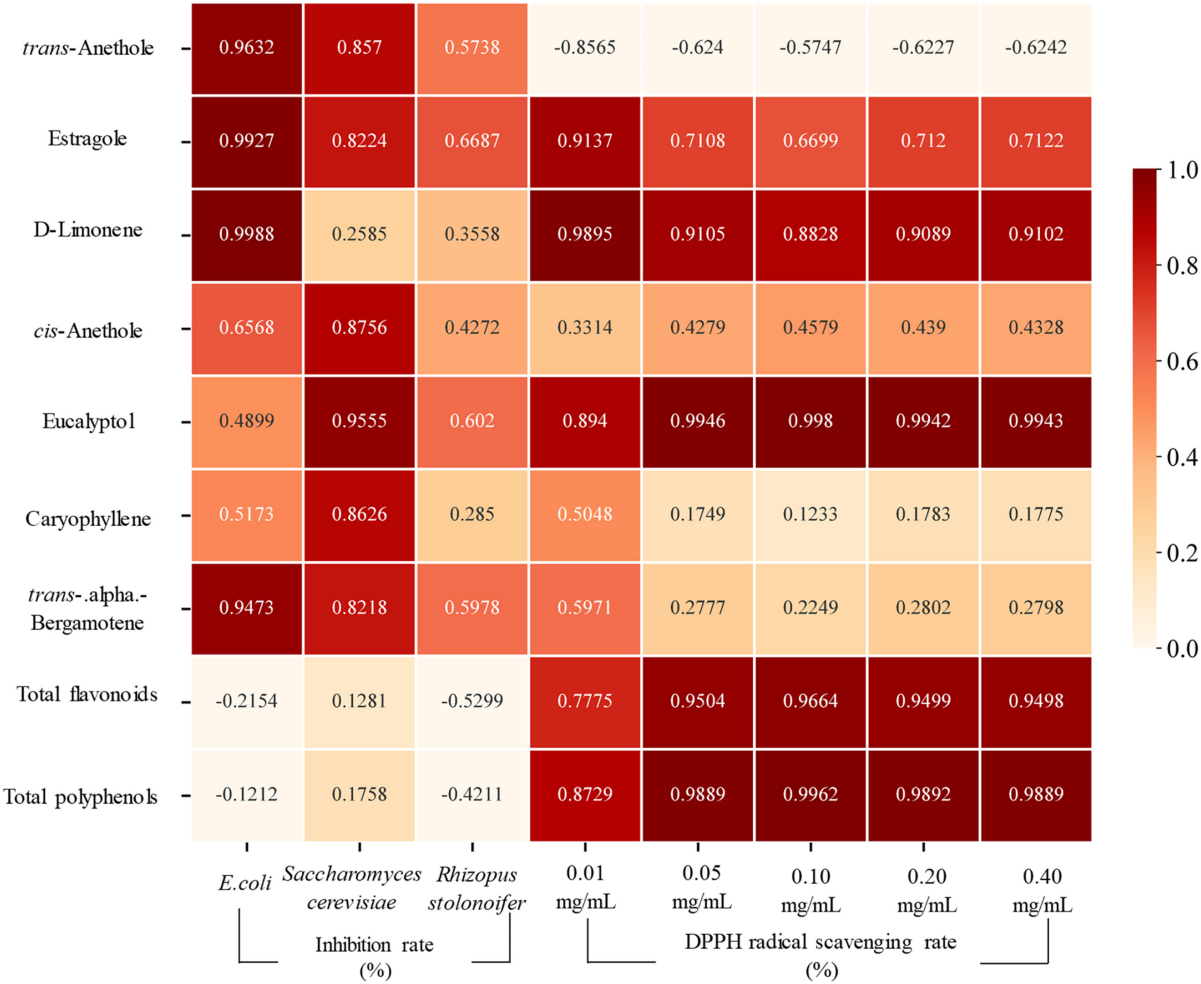
Correlation analysis between the main components and the biological activity of the star anise extracts extracted by different methods. The data in the figure represents the correlation coefficients between the content of major components and biological activities (*r*). The darker of the color indicates the stronger correlation.

Antioxidants can neutralize free radicals and inhibit radical chain oxidation reactions induced by molecular oxygen (Gorinstein et al. 2004). The antioxidant activity of various natural products is often evaluated by using DPPH (Singh et al. 2005). Figure 6D illustrates the DPPH radical scavenging activity of star anise essential oil samples with different concentrations. Vitamin E (VE) exhibited stable and excellent DPPH radical antioxidant activity within the tested range. Compared with other extracts, the star anise essential oils from ethanol Soxhlet extraction presented the best inhibitory effect on DPPH radicals and especially exceeded VE at 0.1, 0.2, and 0.4 mg/mL (*p* < 0.05). The highest inhibition rate of the ethanol extract reported at 1.0 mg/mL was about 80% (Padmashree et al. 2007), but in our study, the inhibition rate at 0.1 mg/mL exceeded 90%, which may have contributed to the variations in raw materials and extraction conditions. The inhibitory effects of steam distillation, supercritical, and subcritical extraction essential oils were all limited. The highest inhibition rate of steam distillation essential oils within the tested concentration range did not exceed 20%, while the reported inhibition rate at the 1.0 mg/mL concentration is about 20%. The difference in the DPPH inhibitory effects among the star anise essential oils extracted with different methods may be related to the variations in the oil compositions. According to Section 3.3, the star anise essential oils extracted from the four methods contain seven common volatile components (d‐limonene, cineole, artemisinin, cis‐anethole, *trans*‐anethole, caryophyllene, and *trans*‐α‐citronelene). The relative contents of these seven volatile components and other nonvolatile active components (total flavonoids and total polyphenols) also differ. As shown in Section 3.6, the antibacterial activity and DPPH scavenging activity both differ among the four types of essential oils. On this basis, the correlations between main component contents and bioactivity were analyzed (Figure 7). Results reveal that the inhibition rates of *E. coli* and *S. cerevisiae* are both closely linked to the relative contents of *trans*‐anethole, artemisinin, and *trans*‐α‐limonene. This finding is consistent with another study (De et al. 2002), indicating the antibacterial activity of star anise is primarily ascribed to anethole. Furthermore, the relative content of d‐limonene is strongly correlated with the inhibition rate of *E. coli*, which is in line with the results of Zhang Yunbin et al. The relative contents of cis‐anethole, cineole, and caryophyllene are also closely related to the inhibition rate of *S. cerevisiae*. Moreover, the DPPH scavenging activity is strongly associated with the relative contents of d‐limonene and cineole, which corroborates two other studies (Juergens et al. 2018; Yu et al. 2019). The contents of total flavonoids and total polyphenol contents are mainly related to the DPPH scavenging activity of essential oils, which is consistent with a report conclusion that the antioxidant activity of star anise essential oils is positively related to the contents of polyphenols and flavonoids (Shobana and Naidu 2000). Moreover, polyphenols in star anise essential oils have antibacterial and antioxidant functions. While all four types of essential oils inhibit *R. stolonifera*, there is no strong correlation between a specific component and the inhibition rate of *R. stolonifera* (Figure 7). This result may be due to the joint participation of multiple components in the inhibition against *R. stolonifera*. Therefore, the difference in the bioactivity of star anise essential oils extracted from different methods is closely related to the oil composition. The antibacterial activity and DPPH scavenging activity of the essential oil result from the collective action of various active ingredients.

### 3.7 Single‐Factor Optimization of Extraction

#### 3.7.1 Single‐Factor Design

**FIGURE 8:**
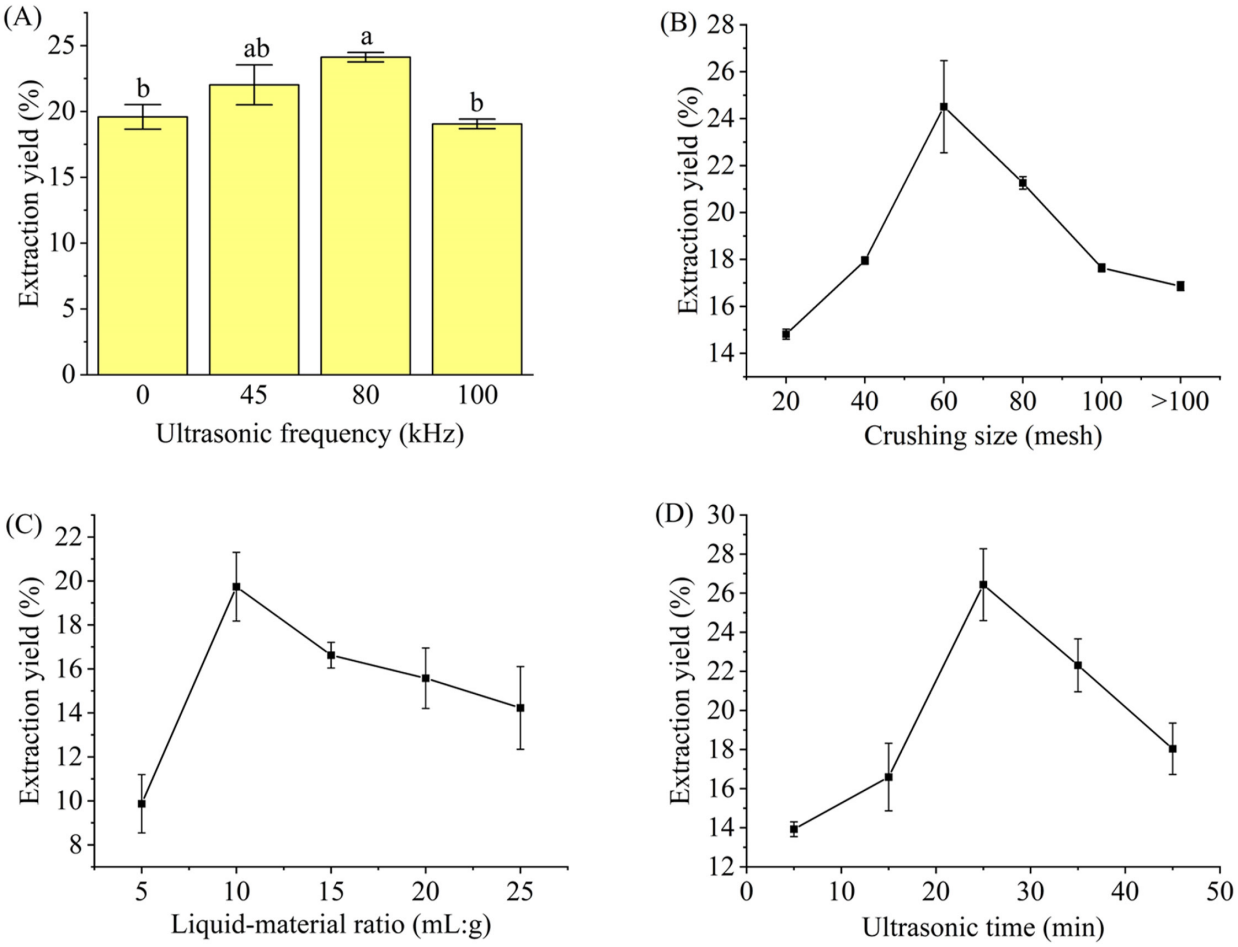
The effects of ultrasonic frequency (A), crushing size (B), liquid–material ratio (C), and ultrasonic time (D) on the yield of star anise extracts.

Based on the results of GC–MS and FT‐IR, the star anise essential oils extracted using the ethanol Figure 8 presents the results of single‐factor optimization with ultrasonic frequency (kHz), ultrasonic time (min), crushing size (mesh), and material–liquid ratio (g:mL). The essential oil yields varied significantly with the ultrasonic frequency, crushing size, material–liquid ratio, and ultrasonic time. While ultrasound‐assisted extraction enhanced the yield of essential oils, a higher ultrasound frequency did not always have a better result from Figure 8A. The yield slightly decreased beyond 80 kHz, which may be caused by more severe cavitation, disturbance, and crushing effects from the higher ultrasound frequency. These effects can disrupt or destroy some unstable components in the essential oils, leading to a reduction in yield (Chaudhari et al. 2021). Thus, an ultrasound frequency of 80 kHz was selected in further extraction. The yield increased with the rise in crushing size within a certain range, as the larger contact area between finer particles and the solvent promoted mass transfer and thorough extraction (Figure 8B). However, the yield decreased beyond 60 meshes, which may be attributed to the increased loss of essential oils due to excessively fine materials (Mandal, Mohan, and Hemalatha 2007). Figure 8C implies that increasing the solvent amount within a certain range facilitates the dissolution of essential oils. However, the material–liquid ratio exceeding 10:1 (mL:g) prolonged the heating and rotary evaporation time, thereby increasing the loss of essential oils and reducing the yield. Furthermore, excessive material–liquid ratio reduced the ultrasound intensity on the materials, thereby decreasing the yield of essential oils. The essential oil yield gradually increased with an extension in ultrasonic time but decreased after 25 min (Figure 8D). The cavitation effect of ultrasound before 25 min made the essential oils dissolve more thoroughly, resulting in an increase in yield. However, excessive ultrasound time resulted in emulsification and loss of unstable components from the essential oils, leading to a decrease in yield. Based on these analyses, the optimal ranges of crushing size, material–liquid ratio, and ultrasonic time were determined as 40–80 meshes, 8–12 (mL:g), and 15–35 min, respectively. These ranges were used as the factor level ranges for further response surface design.

#### 3.7.2 Response Surface Design

Based on the results of the single‐factor analysis, a Box–Behnken design with three factors and three levels was used to optimize the star anise essential oil extraction process. With the yield *Y* (%) as the evaluation index, the coding factors, levels of response surface experiment, and the corresponding results are elaborated in Table 2. Regression fitting and variance analysis were performed using Design‐Expert 8.0.5.

**TABLE 2:** The results of response surface.

No.	A crushing size (mesh)	B material–liquid ratio (mL:g)	C ultrasonic time (min)	*Y* yield (%)
1	80	12	25	18.65
2	60	10	25	24.89
3	40	12	25	17.81
4	40	10	15	19.56
5	60	8	15	17.75
6	60	12	35	20.84
7	60	10	25	25.64
8	60	8	35	18.36
9	60	10	25	25.09
10	60	10	25	27.03
11	40	8	25	15.52
12	60	10	25	26.09
13	80	8	25	17.11
14	80	10	15	19.62
15	60	12	15	16.58
16	40	10	35	19.58
17	80	10	35	23.21

**FIGURE 9:**
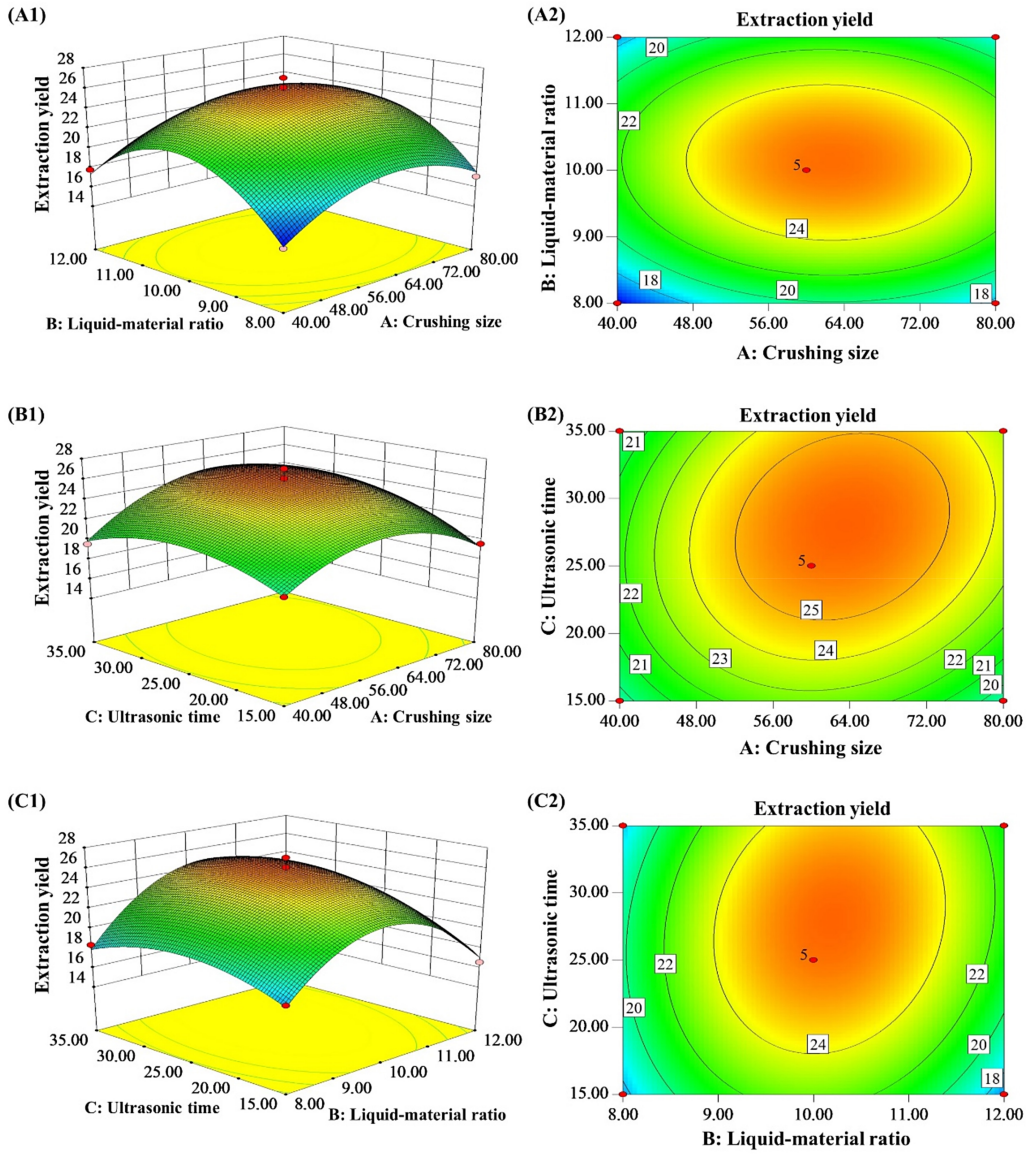
The response surface charts (A1, B1, and C1) and the contour maps (A2, B2, and C2) of the interaction between various influencing factors (A: crushing size; B: liquid–material ratio; and C: ultrasonic time).

Based on the response surface and contour plots in Figure 9 and Table 3, the yield of star anise essential oils increased with the prolonged ultrasonic time and crushing size but was only slightly affected by the material–liquid ratio. Significant interactions were found between ultrasonic time and crushing size and between ultrasonic time and material–liquid ratio. The contours in the contour plots are elliptical, indicating the interactions between two factors are significant. The yield maximizes to 3.23% at ultrasonic time of 28.5 min, crushing size of 65 meshes, and material–liquid ratio of 9.5 (mL:g). The verification experiment confirms the validity of the predicted values, which are very close to the actual yield of star anise essential oils (3.20%). The results suggest the Box–Behnken design combined with response surface methodology is an effective approach for optimizing the ultrasonic‐assisted ethanol extraction process of star anise essential oils.

**TABLE 3:** The ANOVA results.

Source	Sum of squares	Freedom	Mean square deviation	*F*	*p*	Significance
Model	219.57	9	24.40	41.33	< 0.0001	**
A crushing size	4.68	1	4.68	7.93	0.0259	*
B material–liquid ratio	3.30	1	3.30	5.59	0.0499	*
C ultrasonic time	8.99	1	8.99	15.23	0.0059	**
AB	0.14	1	0.14	0.24	0.6404	
AC	3.19	1	3.19	5.40	0.0531	
BC	3.33	1	3.33	5.64	0.0492	*
A^2^	42.65	1	42.65	72.26	< 0.0001	**
B^2^	117.95	1	117.95	199.82	< 0.0001	**
C^2^	18.09	1	18.09	30.65	0.0009	**
Residual	4.13	7	0.59			
Lack of Fit	1.19	3	0.40	0.54	0.6801	
Net error	2.94	4	0.74			
Total	223.70	16				
*R* ^2^ = 0.9815, *R* ^2^_Adj_ = 0.9578						
*Y* = −141.04 + 0.93A + 25.93B + 0.42C−4.69 × 10^−3^AB + 4.46 × 10^−5^ AC + 4.56 × 10^−2^ BC−7.96 × 10^−3^A^2^−1.32B^2^−2.07 × 10^−2^C^2^						

*Note: p* < 0.01 was very significant “**”; *p* < 0.05 was significant “*”.

Figure 9 clearly shows certain interactions among the grinding fineness, liquid‐to‐material ratio, and ultrasonic time. Specifically, the interaction between the liquid‐to‐material ratio and ultrasonic time is significant. When the ultrasonic time is optimal, the liquid‐to‐material ratio more obviously affects the yield. However, reducing or increasing the liquid‐to‐material ratio cannot improve the yield when the ultrasonic time is too short or too long. The reason for this phenomenon is that with insufficient ultrasonic time, the vibration and cavitation effects of ultrasound do not uniformly penetrate the inner layers of the material, and the ultrasound‐assisted mass transfer cannot be fully utilized. When the ultrasonic time is too long, although the ultrasound can fully penetrate the inner and outer layers of the material, it also accelerates the destruction and loss of extracted essential oil components due to emulsification and temperature rise. Based on the optimization analysis of the model using Design‐Expert 8.0.5, the optimal level of each factor was obtained, including grinding fineness of 60 meshes, liquid‐to‐material ratio of 8:1 (mL/g), and ultrasonic time of 35 min. The maximum essential oil yield under these conditions was 25.75%. Three repeated validation experiments were conducted under the optimal conditions, and the average yield was 25.51% ± 0.21%, with a relative error less than 1% from the predicted value. Hence, the response surface model has high predictive accuracy. This result is larger than the star anise essential oil yield of 20.38% obtained by Guo Yuan through ethanol extraction, and is comparable to the essential oil yield of 24.87% obtained through microwave‐assisted alcohol dissolution extraction (Zou et al. 2023). The difference in yields may be related to the extraction method, extraction process conditions (e.g., grinding fineness, liquid‐to‐material ratio, extraction temperature, solvent recovery method), and plant material properties (e.g., variety, origin, maturity).

## 4 Conclusions

The present study is aimed at investigating the influence of extraction methods on the yield, aroma properties, chemical composition, and bioactivity of star anise essential oils. Electronic nose detection reveals that the subcritical extraction essential oils exhibit the most intense aroma, while the aroma of ethanol Soxhlet extraction essential oils is more complex. FT‐IR identifies the essential oil components obtained through various extraction methods, including benzene rings, carbonyl groups, C=C, and aromatic ether bonds. These components mainly comprise heterocyclic olefins, heterocyclic oxygenates, and aromatic oxygenates, as well as certain amounts of flavonoids and polyphenols. Correlation analysis demonstrates strong associations in the relative contents of volatile compounds (e.g., *trans*‐anethole, wormwood, d‐limonene, cineole, *trans*‐α‐citronelene) with the antibacterial activity of the essential oils. Similarly, the contents of d‐limonene and cineole, total non‐volatile flavonoids, and total polyphenols are strongly correlated with the DPPH scavenging activity. The findings emphasize the efficacy of the ethanol Soxhlet extraction method in retaining the bioactivity of essential oils. Optimization experiments using a Box–Behnken central composite design show the optimal extraction conditions are ultrasonic frequency of 80 kHz, crushing particle size of 60 meshes, liquid–material ratio of 8:1 (mL:g), and ultrasonic time of 35 min, which yield 25.51% ± 0.21% essential oils. Overall, this comprehensive study provides valuable insights into the extraction, characterization, and potential applications of star anise essential oils. Further, in order to help ensure proper application and safe use of essential oils as a self‐care modality, the development of a release system for essential oils from antimicrobial packaging or the sustained release of 3D printing functional inks has gained attention. The revision details are highlighted by using red colored text in our resubmitted manuscript.

## Author Contributions

**Guangjie Zhang:** investigation (equal), formal analysis (equal). **Zheng Ma:** data curation (equal). **Yan Piao:** investigation (equal), visualization (equal). **Ning Li:** formal analysis (equal), validation (equal). **Canfeng Bian:** data curation (equal), software (equal). **Chenxi Ren:** data curation (equal), software (equal). **Mingyuan Gao:** investigation (equal), visualization (equal). **Weiming Yue:** validation (equal), supervision (equal). **Tianzhu Guan:** validation (equal), supervision (equal).

## Conflicts of Interest

The authors declare no conflicts of interest.

## Supporting information


Table S1

## Data Availability

All data generated or analyzed during this study are included in this manuscript.
