# Supplementary material for: Revealing the Potential of Star Anise Essential Oil: Comparative Analysis and Optimization of Innovative Extraction Methods for Enhanced Yield, Aroma Characteristics, Chemical Composition, and Biological Activities
Source: Food Sci Nutr. 2024 Oct 18;12(11):9540–54. doi: 10.1002/fsn3.4508 (PMC11606829; doi:10.1002/fsn3.4508)
Supplement: Supplementary file 1 — Table S1 [file FSN3-12-9540-s001.docx]

Supplementary Table 1 The MIC of the star anise essential oil extracted by different methods on three test strains.

| Methods | MIC（mg/mL） | | |
| --- | --- | --- | --- |
|  | *E. coli* | *Saccharomyces cerevisiae* | *Rhizopus stolonoifer* |
| Steam distillation | >6.4 | >6.4 | >6.4 |
| Ethanol soxhlet extraction | >6.4 | >6.4 | 6.4 |
| Supercritical carbon dioxide extraction | >6.4 | >6.4 | 6.4 |
| Subcritical n-butane extraction | >6.4 | >6.4 | >6.4 |

Supplementary Table 2 The coding factors and level of response surface experiment

| Level | A crushing size (mesh) | B material–liquid ratio (mL:g) | C ultrasonic time（min） |
| --- | --- | --- | --- |
| +1 | 80 | 8 | 15 |
| 0 | 60 | 10 | 25 |
| -1 | 40 | 12 | 35 |
